# Should we add unilateral sacrospinous ligament fixation to vaginal hysterectomy in management of stage 3 and stage 4 pelvic organ prolapse?

**DOI:** 10.4274/tjod.93546

**Published:** 2015-09-15

**Authors:** Elif Ağaçayak, Senem Yaman Tunç, Mehmet Sait İçen, Serdar Başaranoğlu, Fatih Mehmet Fındık, Sibel Sak, Yasemin Ceter, Gamze Akın, Talip Gül

**Affiliations:** 1 Dicle University Faculty of Medicine, Department of Obstetrics and Gynecology, Diyarbakır, Turkey; 2 Fatih University Faculty of Medicine, Department of Obstetrics and Gynecology, İstanbul, Turkey; 3 Sedef Medical Center, Clinic of Obstetrics and Gynecology, Diyarbakır, Turkey

**Keywords:** Pelvic organ prolapse, vaginal hysterectomy, sacrospinous ligament fixation

## Abstract

**Objective:**

To compare ‘‘vaginal hysterectomy alone’’ with ‘‘vaginal hysterectomy with prophylactic unilateral sacrospinous ligament fixation’’ in terms of intraoperative complications and 1-year anatomic outcomes and symptoms in patients aged over 50 years who presented with stage 3 or 4 pelvic organ prolapse (POP).

**Materials and Methods:**

Thirty-five patients underwent vaginal hysterectomy alone and 32 patients underwent vaginal hysterectomy with unilateral sacrospinous ligament fixation because of benign pathology between January 2012, and June 2014, were retrospectively analyzed in this study. The patients’ demographic data and preoperative and intraoperative findings were obtained from the hospital records and noted. The patients were invited by phone to a follow-up visit to assess their 1-year anatomic outcomes and symptoms.

**Results:**

There was no significant demographic difference between the patients who underwent vaginal hysterectomy alone and those who had a vaginal hysterectomy with sacrospinous ligament fixation. Both length of operation and hospital stay were significantly longer in the patients who underwent vaginal hysterectomy with sacrospinous ligament fixation (p<0.001); intraoperative complications requiring blood transfusion were also significantly more frequent in these patients compared with the patients who underwent vaginal hysterectomy only (p=0.048). Recurrence of vaginal vault prolapse was significantly more frequent in the patients with vaginal hysterectomy alone compared with those who had both vaginal hysterectomy and sacrospinous ligament fixation (p=0.035).

**Conclusion:**

Unilateral sacrospinous ligament fixation might be added to vaginal hysterectomy in patients with stage 3 or 4 POP who are predicted to have long survival times. However, further studies with a larger sample size are needed in this area of research.

## INTRODUCTION

Pelvic organ prolapse (POP) affects approximately 50% of women aged over 50 years. Its lifetime prevalence is 30-50%. Approximately 11-12% of all women undergo surgery for pelvic floor dysfunction before they reach 80 years of age^(1)^.

Uterovaginal prolapse is a serious health issue that especially occurs in women with a history of vaginal delivery. The incidence of uterovaginal and vaginal vault prolapse is observed to be particularly high among the elderly because pelvic floor structures loosen with age. The etiology of uterovaginal prolapse involves pregnancy, delivery, lifting heavy weights, obesity, increased intra-abdominal pressure because of such factors as pelvic masses, and weakened pelvic floor structures^(2)^. The surgical therapy that is typically adopted in this health issue is vaginal hysterectomy. Various therapeutic techniques have been suggested to be performed at the time of vaginal hysterectomy to avoid potential recurrence of prolapse. However, it remains unclear which of the therapeutic techniques is superior^(3)^.

Several surgical techniques have been suggested to be performed at the time of vaginal hysterectomy to avoid potential recurrence of prolapse. Of these, vaginal techniques are the most frequently adopted because of the following advantages: shorter length of operation, faster healing, and lower rates of adhesion. Sacrospinous ligament fixation (SLF) is one of these techniques^(4,5)^. In brief, SLF refers to suspension of the vaginal vault from the sacrospinous ligament, which extends from the ischial spine, to the coccyx and the lower portion of the sacrum. This technique was first defined by Sederl in 1958. It allows for suspension of the vagina from the sacrospinous ligament, thereby bringing it to a level above the levator ani muscle.

The aim of this study was to compare ‘‘vaginal hysterectomy with prophylactic unilateral SLF’’ with ‘‘vaginal hysterectomy alone’’ in terms of intraoperative complications and 1-year anatomic outcomes and symptoms in patients aged over 50 years who presented with stage 3 or 4 pelvic organ prolapse.

## MATERIALS AND METHODS

This study was conducted in Dicle University Faculty of Medicine, Department of Gynecology and Obstetrics upon provision of ethical approval. Fifty patients who had undergone vaginal hysterectomy alone (VH group) and 50 who had undergone vaginal hysterectomy with SLF (VH+SLF group) because of benign pathologies between January 2012, and June 2014, were retrospectively analyzed. Patients aged 50 years and older and staged as stage 3-4 POP according to the POPQ classification system were included in the study (Table 1). Patients with stage 1-2 POP, those who had connective tissue diseases, cancer, immobility, and massive obesity were excluded from the study.

Obesity-BMI ≥30 kg/m^2^; class 1-BMI of 30.0 to 34.9; class 2-BMI of 35.0 to 39.9 kg/m^2^; and class 3-BMI ≥40 kg/m^2^. This type of obesity is also referred to as severe, extreme, or massive obesity^(6)^. The patients’ demographic data and preoperative and intraoperative findings were obtained from the hospital records. Data on reasons for presentation to hospital, age at operation, vaginal parity, medical problems, menopausal status, history of operations, stage of prolapse, type and length of operation, length of hospital stay, intraoperative complications (bleeding that required blood transfusion, rectal and bladder injuries, febrile morbidity, and nerve injuries), and 1-year anatomic outcomes (vaginal vault prolapse, cystocele, rectocele, dyspareunia, chronic constipation, abdominal pain and urinary incontinence) were noted. The patients were requested to visit the hospital for follow-up in postoperative week 6 and at 1 year. No-show patients were called by phone and invited to the hospital such that we could assess their 1-year surgical outcomes. Two (4%) patients in the VH group and 3 (6%) from the VH+SLF had died in the meantime of various systemic diseases. Furthermore, 9 (18%) VH patients and 13 (26%) VH+SLF patients could not be reached by phone. In addition, 4 (8%) VH patients and 2 (4%) VH+SLF patients stated that they would not be able to come to the follow-up visit because of long journey distances to the hospital. In total, 33 patients were lost to follow-up, and the remaining 67 patients with 1-year follow-up data were included in the study (35 VH patients and 32 VH+SLF patients).

A total of 67 postmenopausal patients with stage 3 (the most distal portion of the prolapse protrudes more than 1 cm below the hymen but no farther than 2 cm less than the total vaginal length) or 4 (vaginal eversion is essentially complete) pelvic organ prolapse were included in this study. POP staging was done according to POPQ classification system (Table 1)^(7)^. Points and landmarks for POPQ system examination. Aa; point A anterior, Ap; point A posterior, Ba; point B anterior; Bp; point B posterior; C; cervix or vaginal cuff, D; posterior fornix (if cervix is present), gh; genital hiatus, pb; perineal body, tvl; total vaginal length. Indications for hysterectomy were identified using the criteria defined by Dicker^(8)^.

### Surgical technique

The patients were reexamined under anesthesia in a lithotomy position after cleansing the surgical site and positioning sterile drapes. The patients then underwent vaginal hysterectomy, which was followed by a preliminary repair for stage 3 or 4 uterovaginal prolapse. The patients with stress incontinence additionally underwent transobturator tape (TOT) procedures. Then, unilateral SLF was performed (using the technique defined by Nichols)^(4)^ as follows: after the rectovaginal space was opened to the vaginal apex, the right pararectal space was entered using blunt dissection; the ischial spine was palpated and taken as the reference to pinpoint the sacrospinous ligament, which extends from the ischial spine medially to the coccyx and the lower portion of the sacrum. The pararectal fascia was penetrated, and the space was enlarged using blunt dissection; the rectum was retracted to the left using two Breisky-Navratil retractors, thereby exposing the sacrospinous ligament. No 1 non-absorbable suture (Prolene) was placed 2-2.5 cm medially to the ischial spine, and one end of the suture was passed through the vaginal vault; surplus tissue located in the posterior vaginal wall was excised, and the upper 1/3 of the vaginal mucosa was repaired. Following the vaginal vault repair, the vaginal vault was suspended from the right sacrospinous ligament by tying together the sacrospinous sutures located proximal to the apex of the vaginal vault. Lastly, posterior repair and perineoplasty were performed, which marked the end of the procedure.

The decision of adding SLF or not to the vaginal hysterectomy was made according to the criteria used by Cruikshank et al.^(9)^ total prolapse uterosacral-cardinal ligament, descent of the vaginal apex to the introitus or lower when pulled after hysterectomy and total plastic operations, and the presence of total procedentia. All surgeries were performed by expert physicians.

### Statistical analysis

All statistical analyses were performed using SPSS version 11.5. Chi-Square test and Fischer’s Exact test (2x2 Tables) were used for comparison of categorical variables. Student’s t-test was used for comparison of continuous variables that demonstrated normal distribution, and Mann-Whitney U test was used for comparison of those that did not demonstrate normal distribution. Data were expressed in mean + standard deviation. A p value smaller than 0.05 was considered statistically significant.

## RESULTS

A total of 67 patients were included in this study; 35 (70%) underwent vaginal hysterectomy alone and 32 (64%) patients underwent vaginal hysterectomy with SLF. The patients’ demographic data are shown in Table 2. There were no statistically significant differences between the two groups in age, parity, sexual activity, medical problems, and reasons for presentation to hospital. All of the patients were postmenopausal. Data on preoperative stages of prolapse are reported in Table 3. Both preoperative uterine prolapse + cystocele and preoperative 3^rd^ degree cystocele were significantly more frequent in the VH group (p=0.01 and p=0.015, respectively). The frequency of preoperative 3^rd^ degree rectocele was significantly higher in the VH+SLF group (p=0.006). Anterior colporrhaphy was significantly more frequent in the VH group (p=0.038), whereas posterior colporrhaphy was significantly more frequent in the VH+SLF group (p=0.017). Comparisons between the groups in terms of type, length of operation, and length of hospital stay are demonstrated in Table 4. Both length of operation and hospital stay were significantly longer in the VH+SLF group (p=0.000). Intraoperative complications are shown in Table 5. In this respect, bleeding that required blood transfusion was significantly more frequent in the VH+SLF group (p=0.048). One-year complications were documented in Table 6. Of which, recurrence of vaginal vault prolapse was significantly more frequent in the VH group (p=0.035).

## DISCUSSION

In this study, intraoperative complications and 1-year anatomic outcomes and symptoms were investigated in patients who underwent vaginal hysterectomy alone as well as in patients who underwent vaginal hysterectomy with SLF. The purpose of the study was to provide an answer to the question ‘‘Should we add prophylactic unilateral SLF to vaginal hysterectomy in management of patients with stage 3 or 4 pelvic organ prolapse?’’ Several studies in the literature have investigated the outcomes of SLF; however, the literature lacks studies that compare vaginal hysterectomy alone with vaginal hysterectomy with SLF.

In a previous study, the authors compared SLF with total mesh and found higher blood loss in patients who underwent SLF^(10)^.Colombo and Milani compared SLF with McCall culdoplasty and found that the latter yielded better results in terms of length of operation, blood loss, and recurrence of prolapse^(11)^. In addition, Maher et al.^(12)^ compared iliococcygeus fixation with SLF and found similar results in the two groups of patients in terms of gluteal pain, hemorrhage, and recurrence of prolapse. In agreement with the literature, the present study demonstrated that patients who underwent VH+SLF had significantly higher blood loss compared with those who underwent vaginal hysterectomy alone. Significantly higher blood loss in SLF might be explained by the fact that the surgical site is narrow, and the hypogastric venous plexus and the pudendal vasculature are located in proximity to the sacrospinous ligament. In this respect, firm vaginal tampons, arterial ligation, and hemoclips might be considered in management of hemorrhage^(13)^. In the present study, 6 (18.8%) of the women who had VH+SLF had hemorrhage. In one (3.1%) of these patients, hemorrhage occurred in the obturator vessels during the TOT procedure. The patients with hemorrhage received spongostan. The content of 2 ampoules of solution was poured on gauze soaked with Transamin 5%, and tamponation was performed.

In the present study, one (3.1%) of the VH+SLF patients experienced perineal nerve injury, which resulted in drop foot, because of surgical positioning. This patient was referred to the department of physical therapy and rehabilitation. The patient’s first-year follow-up revealed that impairment of pain sensation in the foot was minimal. The sciatic nerve and the its branching perineal nerves can be damaged during lengthy vaginal operations because of surgical positioning. Moreover, healing might be quite slow with such damage^(14)^.

In the present study, there was no significant difference between the two groups in terms of patient satisfaction with their operations. In agreement with the literature, SLF following vaginal hysterectomy did not cause additional symptoms and dissatisfaction in the patients compared with vaginal hysterectomy alone(15). Furthermore, recurrence of vaginal vault prolapse was significantly more frequent in the VH group compared with the VH+SLF group. However, there was no significant difference between the two groups in cystocele and rectocele recurrence. Previous studies reported that the risk of cystocele recurrence increased in unilateral patients undergoing SLF, not that of apical prolapse^(16,17,18)^. In a study in which patients with POP underwent SLF and followed up at one and seven years, the objective cure rate for apical vaginal vault prolapse at 1 and 7 years was 96% (49/51) and 94.28% (33/35), respectively^(19)^. In the present study, of all the patients who underwent vaginal hysterectomy with SLF, 1 patient (3.1%) had recurrence of vaginal vault prolapse, 5 (15.6%) had cystocele recurrence, and 1 (3.1%) had rectocele recurrence. Unlike the results of the previous studies, there was no significant difference between the two groups in postoperative cystocele formation, which might have been caused by the relatively lower degrees of cystocele that the VH+SLF group had preoperatively. The frequency of preoperative 3^rd^ degree cystocele was significantly higher in the VH group compared with the VH+SLF group.

Patients who undergo unilateral SLF may experience urinary dysfunction. However, this usually happens when the procedure is accompanied by anterior colporrhaphy or Burch colposuspension^(20,21)^. In the present study, 12 (34.3%) VH patients and 4 (12.5%) VH+SLF patients underwent anterior colporrhaphy. In this respect, the frequency of anterior colporrhaphy was significantly higher in the VH group.

Many studies have investigated the relationship of POP and stress incontinence. One study showed that 15-80% of patients with POP have stress incontinence^(22)^. Although the relationship with stress incontinence is known, patients with POP rarely report stress incontinence. Some of these women may have stress incontinence but they are clinically continent^(23)^. Rosenzweig et al.^(24)^ reported a 59% rate of occult urodynamic stress incontinence when a simple pessary was used to correct the prolapses. Similarly, Ghoeneim et al.^(25)^ showed that the use of a pessary lead to 68% occult urodynamic stress incontinence. In the present study, similar to the studies outlined above, occult stress urinary incontinence was found in the preoperative stress test performed using a pessary. For this reason, 20 (57.1%) VH patients and 12 (37.5%) VH+SLF patients additionally underwent TOT procedures. In this respect, there was no significant difference between the groups. Although the frequency of anterior colporrhaphy was significantly higher in the VH group, there was no significant difference between the groups in urinary dysfunction, which may have resulted from the study’s small sample size.

Previous studies in the literature concluded that SLF was an inexpensive and safe method that could be used in the management of advanced-stage pelvic organ prolapse. In addition, they listed the long-term complications of SLF as follows: gluteal pain, back pain, inguinal pain, and de novo urinary incontinence^(13)^. In another study, major intra-and postoperative complications not occurred the long-term complications of SLF as follows^(26)^. In the present study, 7 (21.9%) patients had gluteal pain, 3 (9.4%) had chronic constipation, and 1 (3.1%) had urinary incontinence as revealed by the 1-year follow-up. In addition, 1 patient (3.1%) had vaginal vault dehiscence and evisceration. There was no significant difference between the groups in 1-year complications. The patient who had intestinal dehiscence underwent vaginal vault repair under local anesthesia.

In their study, Given et al.^(27)^ suggested that the vagina became shorter after SLF, which possibly caused dyspareunia. In a previous study, the authors indicated an association between dyspareunia and posterior colporrhaphy and prenioplasty in patients who underwent SLF^(28)^. Lopes et al.^(29)^ compared mesh with SLF and found that sexual dysfunction was significantly more likely in the mesh group. In the present study, there was no significant difference between the two groups in sexual dysfunction. In addition, no isolated dyspareunia was observed in the VH+SLF group. However, 1 patient (3.1%) in this group reported dyspareunia accompanied by abdominal pain. In this respect, there was no significant difference between the groups in experience of dyspareunia. These results might be explained by the small sample size of the study because only 18 patients (51.4%) in the VH group and 14 (43.8%) in the VH+SLF group were sexually active.

The mean length of operation was 99.2±29.6 min in the VH+SLF group, which is relatively short compared with similar studies in the literature. In addition, the length of operation was significantly longer in the VH+SLF group compared with the VH group. The relatively short mean length of operation may be because the surgeons of Dicle University Hospital, which serves as a tertiary care center, have considerable surgical experience with stage 3 and 4 POP because this condition is quite widespread among women of the region who are very much involved in agricultural activities. On the other hand, length of hospital stay was 3.9±2.5 days in the VH+SLF group, which was significantly higher than that in the VH group. Similar studies in the literature reported shorter length of hospital stay^(30)^. This result may be explained by the fact that all of the study participants were postmenopausal elderly patients.

Anterior pelvic plane meshes are recommended to secured to the sacrospinous ligament safely during the SLF operation^(31)^ Mesh was not used in any of our patients.

In conclusion, vaginal hysterectomy with SLF increases the length of operation and hospital stay as well as the risk of intraoperative and early complications in patients with stage 3 or 4 POP. However, recurrence of vaginal vault prolapse was significantly more frequent after vaginal hysterectomy alone. Given all these, unilateral SLF may be added to vaginal hysterectomy in patients with stage 3 or 4 POP who are predicted to have long survival times. However, further studies with a larger sample sizes are needed in this area of research.

## Figures and Tables

**Table 1:**
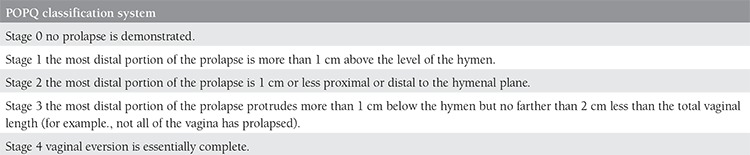
POPQ classification system

**Table 2:**
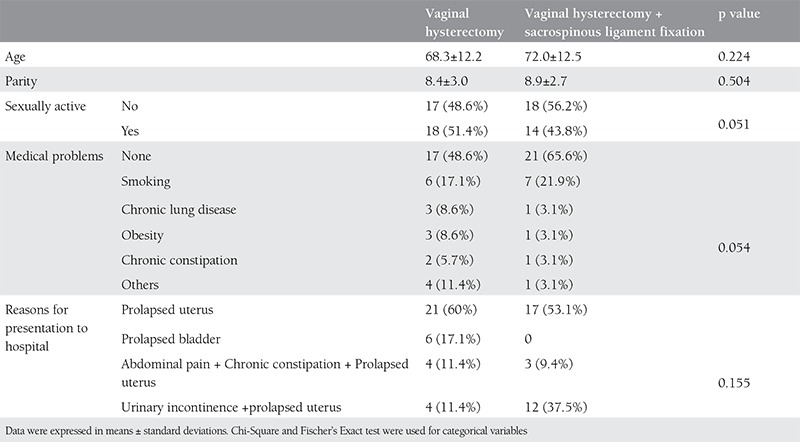
Patients’ demographic data

**Table 3:**
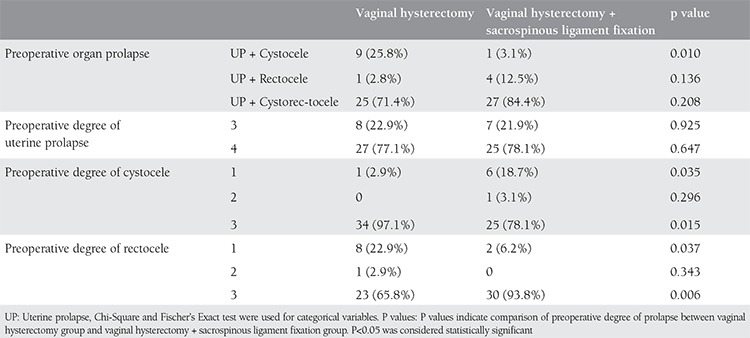
Preoperative degree of prolapse

**Table 4:**
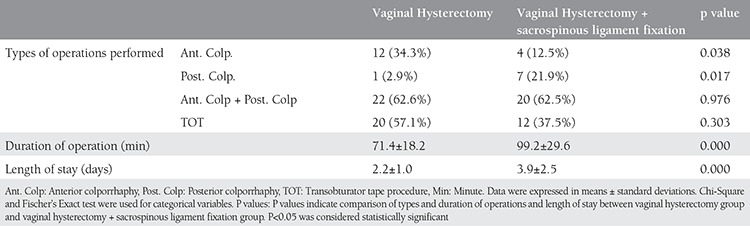
Types and duration of operations and length of stay

**Table 5:**
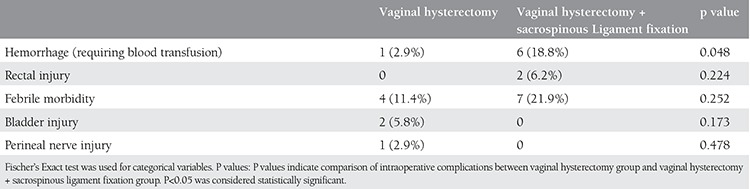
Intraoperative complications

**Table 6:**
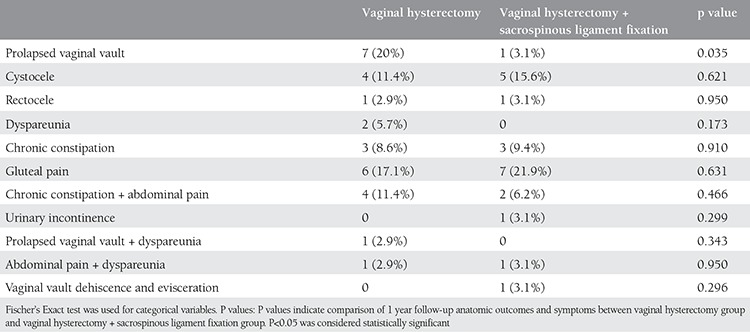
One year follow-up anatomic outcomes and symptoms

## References

[1] Nygaard I, Barber MD, Burgio KL, Kenton K, Meikle S, Schaffer J, et al (2008). for the Pelvic Floor Disorders Network. Prevalence of symptomatic pelvic floor disorders in US women. JAMA.

[2] Chou LY, Chang DY, Sheu BC, Huang SC, Chen SY, Chang WC (2010). Clinical out-come of transvaginal sacrospinous fixation with the Veronikis ligature carrier in genital prolapse. Eur J Obstet Gynecol Reprod Biol.

[3] Dökmeci F (2005). Therapeutic options in pelvic organ prolapse and urinary incontinence. Turkiye klinikleri j ınt Med Sci.

[4] Randall CL, Nichols DH (1971). Surgical treatment of vaginal inversion. Obstet Gynecol.

[5] Imparato E, Aspesi G, Rovetta E, Presti M (1992). Surgical management and prevention of vaginal vault prolapse. Surg Gynecol Obstet.

[6] No authors listed (2000). Obesity: preventing and managing the global epidemic. Report of a WHO consultation. World Health Organ Tech Rep Ser.

[7] Bump RC, Mattiasson A, Bo K, Brubaker LP, DeLancey JO, Klarskov P, et al (1996). The standardization of terminology of female pelvic organ prolapse and pelvic floor dysfunction. Am J Obstet Gynecol.

[8] Dicker RC, Greenspan JR, Strauss LT, Cowart MR, Scally MJ, Peterson HB, et al (1982;1). Complications of abdominal and vaginal hysterectomy among women of reproductive age in the United States. The Collaborative Review of Sterilization. Am J Obstet Gynecol.

[9] Cruikshank SH, Cox DW (1990). Sacrospinous ligament fixation at the time of transvaginal hysterectomy. Am J Obstet Gynecol.

[10] Duran B, Koç Ö, Topçuoğlu A, Eşitken C (2011). Comparison of perioperative complications and long-term consequences in vaginal sacrospinous ligament fixation, abdominal sacrocolpopexy and total mesh procedures abant izzet baysal. Journal of Faculty of Medicine.

[11] Colombo M, Milani R (1998). Sacrospinous ligament fixation and modified McCall culdoplasty during vaginal hysterectomy for advanced uterovaginal prolapse. Am J Obstet Gynecol.

[12] Maher CF, Murray CJ, Carey MP, Dwyer PL, Ugoni AM (2001). Iliococcygeus or sacrospinous fixation for vaginal vault prolapse. Obstet Gynecol.

[13] Cruikshank SH, Muniz M (2003). Outcomes study: a comparison of cure rates in 695 patients undergoing sacrospinous ligament fixation alone and with other site specific procedureda 16-year study. Am J Obstet Gynecol.

[14] Burkhart FL, Daly JW (1966). Sciatic and Peroneal Nerve Injury: A Complication of Vaginal Operations. Obstet Gynecol.

[15] Peng P, Zhu L, Lang JH, Wang WY, Shi HH (2010). Unilateral sacrospi-nous ligament fixation for treatment of genital prolapse. Chin Med J (Engl).

[16] Sze EH, Karram MM (1997). Transvaginal repair of vault prolapse: a review. Obstet Gynecol.

[17] Cundiff GW (1998). Management of pelvic organ prolapse. Obstet Gynecol Clin North Am.

[18] Dogan E, Demir N, Altınyurt S, Alper Ç, Güçlü S, Saygılı U (2003). sacrospinous fixation during vaginal hysterectomy. Türkiye Klinikleri J Gynecol Obst.

[19] Aksakal O, Doğanay M, Onur Topçu H, Kokanali K, Erkilinç S, Cavkaytar S (2014). Long-term surgical outcomes of vaginal sacrospinous ligament fixation in women with pelvic organ prolapse. Minerva Chir.

[20] Lantzsch T, Goepel C, Wolters M, Koelbl H, Methfessel HD (2001). Sacrospinous ligament fixation for vaginal vault prolapse. Arch Gynecol Obstet.

[21] Nichols DH (1982). Sacrospinous fixation for massive eversion of the vagina. Am J Obstet Gynecol.

[22] Borstad E, Rud T (1989). The risk of developing urinary stres incontinence after vaginal repair in continent women. A clinical and urodynamic fallow-up study. Acta Obstet Gynecol Scand.

[23] Bump RC, Fantl JA, Hurt WG (1988). The mechanism of urinary continence in women with severe uterovaginal prolapse;results of barrier studies. Obstet Gynecol.

[24] Rosenzweing BA, Pushkin S, Blumenfeld D, Bhatia NN (1992). Prevalence of abnormal urodynamics test result in continent women with severe genitourinary prolapse. Obstet Gynecol.

[25] Ghoneim GM, Walters F, Lewis V (1993). The value of the vaginal pack test in large cystoceles. J Urology.

[26] Gupta P (2015). Transvaginal sacrospinous ligament fixation for pelvic organ prolapse stage III and stage IV uterovaginal and vault prolapse. Iran J Med Sci.

[27] Given Jr FT, Muhlendorf IK, Browning GM (1993). Vaginal length and sexual function after colpopexy for complete uterovaginal eversion. Am J Obstet Gynecol.

[28] Holley RL (1996). Sexual dysfunction after sacrospinous ligament fixation. J Reprod Med.

[29] Lopes ED, Lemos NL, Carrama˜o Sda S, Lunardelli JL, Ruano JM, Aoki T, et al (2010). Transvaginal polypropylene mesh versus sacrospinous ligament fixation for the treatment of uterine prolapse: 1-year follow-up of a randomized controlled trial. Int Urogynecol J.

[30] Öktem M, Eroğlu D, Esinler İ, Başer E, Yanık F, Kuşçu E, et al (2007). Transvaginal sacrospinous ligament fixation technique as part of the vaginal repair procedure for marked uterovaginal prolapse. Turk j Obstet Gynecol.

[31] Menahem N, Natalia S, Vladimir S, Jacob B (2014). Anterior needle-guided mesh in advanced pelvic organ prolapse: apical fixation on sacrospinous ligaments. Eur J Obstet Gynecol Reprod Biol.

